# Short-Term Effects of Botulinum Toxin-A Injection on the Medial Gastrocnemius Histological Features in Ambulant Children with Cerebral Palsy: A Longitudinal Pilot Study

**DOI:** 10.3390/toxins16020069

**Published:** 2024-01-30

**Authors:** Jorieke Deschrevel, Anke Andries, Karen Maes, Nathalie De Beukelaer, Marlies Corvelyn, Lauraine Staut, Hannah De Houwer, Domiziana Costamagna, Kaat Desloovere, Anja Van Campenhout, Ghislaine Gayan-Ramirez

**Affiliations:** 1 Laboratory of Respiratory Diseases and Thoracic Surgery, Department of Chronic Diseases and Metabolism, O&N 1bis Box 706, Herestraat 49, 3000 Leuven, Belgium; jorieke.deschrevel@kuleuven.be (J.D.); anke.andries@kuleuven.be (A.A.); karen.maes@kuleuven.be (K.M.); 2 Neurorehabilitation Group, Department of Rehabilitation Sciences, Tervuursevest 101 Box 1501, 3000 Leuven, Belgium; nathalie.debeukelaer@kuleuven.be (N.D.B.); lauraine.staut@kuleuven.be (L.S.); domiziana.costamagna@kuleuven.be (D.C.); kaat.desloovere@kuleuven.be (K.D.); 3 Stem Cell and Developmental Biology, Department of Development and Regeneration, O&N4 Box 804, 3000 Leuven, Belgium; marliescorvelyn@hotmail.com; 4 Pediatric Orthopedics, Department of Development and Regeneration, Herestraat 49 Box 7003, 3000 Leuven, Belgium; hannah.dehouwer@gmail.com (H.D.H.); anja.vancampenhout@uzleuven.be (A.V.C.); 5 Exercise Physiology Research Group, Department of Movement Sciences, Tervuursevest 101 Box 1500, 3000 Leuven, Belgium

**Keywords:** microbiopsy, myosin heavy chain, spasticity, muscle fiber size, muscle fiber proportion, satellite cells, capillaries, coefficient of variation, botulinum toxin-A, muscle integrity

## Abstract

Botulinum toxin-A (BoNT-A) injection is known to exert beneficial effects on muscle tone, joint mobility and gait in children with cerebral palsy (CP). However, recent animal and human studies have raised the concern that BoNT-A might be harmful to muscle integrity. In CP-children, the impact of BoNT-A on muscle structure has been poorly studied, and inconsistent results have been reported. This study was aimed at determining the time course effect of a single BoNT-A administration on medial gastrocnemius (MG) morphology in CP-children. MG microbiopsies from 12 ambulant and BoNT-A-naïve CP-children (age, 3.4 (2.3) years, ranging from 2.5 to 7.8 years; seven boys and five girls; GMFCS I = 5, II = 4 and III = 3) were collected before and 3 and 6 months after BoNT-A treatment to analyze the fiber cross-sectional area (fCSA) and proportion; capillarization; and satellite cell (SC) content. Compared with the baseline, the fCSA decreased at 3 months (−14%, NS) and increased at 6 months (+13%, NS). Fiber size variability was significantly higher at 3 months (type I: +56%, *p* = 0.032; type IIa: +37%, *p* = 0.032) and 6 months (type I: +69%, *p* = 0.04; type IIa: +121%, *p* = 0.032) compared with the baseline. The higher type I proportion seen at 3 months was still present and more pronounced at 6 months (type I: +17%, *p* = 0.04; type IIx: −65%, *p* = 0.032). The capillary fiber density was reduced at 3 months (type I: −43%, NS; type II: −44%, *p* = 0.0320) but normalized at 6 months. There was a non-significant increase in SC/100 fibers at 3 months (+75%, NS) and 6 months (+40%, NS) compared with the baseline. These preliminary data suggest that BoNT-A induced alterations in the MG of children with CP, which were still present 6 months after BoNT-A injection but with signs of muscle recovery.

## 1. Introduction

Cerebral palsy (CP) is the most common cause of childhood disability, affecting 1.6 per 1000 live births in high-income countries [1]. CP is caused by a non-progressive upper motor neuron lesion in the developing or immature brain, resulting in progressive neuromuscular problems [2,3]. Spasticity, defined as the velocity-dependent resistance to stretch [4], is the most common symptom in CP, affecting 77–86% of children [5,6]. It occurs because of a loss of inhibition in the spindle stretch reflex caused by the upper motor neuron lesion, leading to “hyperreflexia” and increased muscle tone [7]. Consequently, this limits the range of motion and contributes to contractures, and posture and movement disorders [3,5,8].

Local botulinum neurotoxin-A (BoNT-A) injections stand as the standard first-line treatment for spasticity in many countries [9]. BoNT-A, which is a neurotoxin obtained from *Clostridium botulinum* bacteria, impedes the exocytosis of acetylcholine (ACh) in the synaptic cleft at the neuromuscular junction, thereby obstructing the release of ACh [10]. The botulinum toxin is widely used as a spasticity management treatment in children with cerebral palsy. It is mostly used in younger children (2 to 8 years) when gait is not fully mature and during growth to prevent contracture formation and to improve gait [9]. In certain indications, this can be followed by a more permanent treatment for spasticity reduction, such as selective dorsal rhizotomy or intrathecal baclofen pump. Furthermore, these children, especially in cases of more generalized spasticity, often also receive oral medication (for example, oral baclofen). The beneficial effects of BoNT-A treatment in ambulant children with CP include decreased muscle tone accompanied by improved joint mobility and muscle function, especially gait amelioration [9].

However, multiple studies on animals have raised concerns that BoNT-A might alter muscle integrity. Indeed, apart from muscle mass loss in different lower limb muscles of rabbits [11,12,13] and rats [14,15,16,17,18,19,20] regardless of whether the dose of BoNT-A was low (3.5 U/kg) [11,12,13,18,19,21] or high (6 U/kg or higher) [14,15,16,17,18,19,20,22,23], alterations in muscle composition have been reported after BoNT-A injection, whatever the dosage. These include decreased contractile elements [12,17], an excess of non-contractile tissue [11,12,13,14,15,17], increased fiber grouping [14] and a shift toward a slower profile [14,15,18,22]. This is associated with a reduction in muscle force [13,14,15,18] that persists up to 6 [13] and 12 [15] months after BoNT-A injection. These studies also show that muscle structural alterations (mass; fiber size and shift) were still present at 3-months post-high-dose BoNT-A [15,20] or 6-months post-BoNT-A injections with both low and high doses [11,13,15] but recovered after 12 months with high dose [15]. This was not the case for muscle function, which remained depressed for 12 months after BoNT-A injection [15]. Gene expression studies have shown the mRNA upregulation of fibrosis-related markers [12,17], markers implicated in muscle metabolism [12,17,21] and inflammation [17,21] up to 6-months post-BoNT, whatever the dose. Additionally, a micro-array study following a single high dose of BoNT-A showed severe transcriptional adaptation, leading to muscle atrophy and weakness one-week post-BoNT, followed by the activation of the extracellular matrix (ECM) and fibrosis-related genes at 4 weeks [23]. This transcriptome was shown to return to a baseline state after 12 weeks [23]. Although these animal studies showed impaired muscle integrity and alterations in several molecular pathways after BoNT-A injection, caution is needed when interpreting these data. In particular, the doses of BoNT-A used in these animal studies were often high (dosages commonly varied between 3 and 6 U/Kg in rats [14,15,16,18] and rabbits [11,12,13,21]); the type of muscles injected varied between studies (quadriceps, tibialis anterior, triceps surae); and the intervals between repeated injections were short (monthly or every 3 months [11,12,13,14,21]) compared with the treatment regimen used in children with CP. Moreover, some studies have compared the impact of treatment with different (increasing) dosages with ranges of up to 10 or 18 U/kg [18,19], even further exceeding the commonly applied dosages used in children with CP.

Intriguingly, in healthy volunteers, MRI analysis has indicated a reduction in the medial gastrocnemius (MG) cross-sectional area 3 months after a single injection with BoNT-A [24] and in the facial procerus volume 1 month after the injection [25]. These reductions were still present one year after the injection [24]. In addition, histological analysis has revealed that the fiber cross-sectional area (fCSA) of the MG measured at one-year post-BoNT-A was reduced by 24% compared with a saline-injected muscle specimen [24]. Moreover, the persistence of muscle atrophy and weakness was reported in the flexor digitorum superficialis and profundus muscles of musicians with focal hand dystonia up to 3.5 years after the cessation of a multiple-BoNT-injection therapy compared with healthy controls [26]. In children with CP, MRI data have also shown a decreased volume in the MG, soleus, tibialis anterior and hamstring after BoNT-A injection compared with the baseline data, and this decrease was still present 6-months post-BoNT-A injection [27]. Along the same line, compared with the baseline condition, 3D ultrasound imaging of the MG revealed a reduced cross-sectional growth 6-months post-BoNT-A, as well as a lower volume growth rate in children with CP [28,29]. Moreover, in children with unilateral spastic CP, the lean mass of the affected leg was shown to be decreased at 4 weeks after BoNT-A injection and to recover after 12 weeks in comparison with the non-involved leg [30].

To the best of our knowledge, at the histological levels, only two studies have analyzed muscle morphological alterations after BoNT-A injections in children with CP [31,32], but they showed contradictory results. While a decrease in fiber size was found in the MG muscle 4 months to 3 years after BoNT-A injection compared with the vastus lateralis muscle, larger fibers were reported in different upper arm muscles of BoNT-A-treated children with CP 5 months to 4 years after treatment compared with BoNT-A-naïve children with CP [31]. There was also a loss of type I fibers with a predominance of type II fibers in the gastrocnemius muscle, which was found to be strongly correlated with the number of BoNT-A injections [32]. In upper arm muscles, larger fibers after BoNT-A were associated with increased capillarization and other pathological signs, including central nuclei, neonatal myosin heavy chain expression, angular fibers and hybrid fibers [31]. Inconsistent data may be related to the fact that the effects of BoNT-A might differ between upper and lower limb muscles. Comparison with age-matched typically developing children and/or with control data for the same muscle type would probably help to better delineate the effects of BoNT-A on muscle integrity in children with CP and to explore whether or not these alterations, if any, are reversible. 

Therefore, this pilot study aimed at determining the time course of the structural alterations in the MG muscle in children with CP following a single BoNT-A injection. Microbiopsies of the MG muscle were collected in ambulant children with CP before and 3 months and 6 months after the first BoNT-A injection to determine fiber size and proportion and the number of capillaries and satellite cells. Because the current pilot study is characterized by rather limited sample sizes, with some missing data at 3-months post-BoNT-A, the main focus will be on the comparison between baseline and 3-months post-BoNT-A and between baseline and 6-months post-BoNT-A, while the potential recovery between 3-months and 6-months post-BoNT will be qualitatively described. We hypothesized (1) that we would find denervation-induced structural alterations 3 months after BoNT-A injection and (2) that denervation-induced structural alterations would still be present 6 months after BoNT-A treatment but with signs of muscle recovery.

## 2. Results

### 2.1. Population Characteristics

The population consisted of 12 ambulant children with CP who were BoNT-A-treatment-naïve at baseline (age 3.4 (2.3) years, ranging from 2.5 to 7.8 years; seven boys and five girls; GMFCS I = 5, II = 4 and III = 3). The modified Ashworth score (MAS) of the medial gastrocnemius from the enrolled children with CP varied between 1 and 3 (five children had MAS 3; three children had MAS 2; two children had MAS 1+; one child had MAS 1; and there was one missing MAS value). Biopsies were collected from all children at baseline and 6-months post-BoNT-A and from 9/12 children 3 months after BoNT-A injection. All children received BoNT-A injections in the MG, with a dosage ranging between 1 and 3 units (Botox^®^, Allergan) per kilogram of body weight. Apart from the injections in the MG, the majority of the children also received BoNT-A injections in other lower limb muscles, namely, in the medial hamstrings (8/12, dosage range of 1–3 units per kilogram of body weight), the gracilis (7/12, dosage range of 1–2 units per kilogram of body weight), hip adductors (4/12, dosage range of 1–2 units per kilogram of body weight), the psoas (2/12, dosage range of 1–2 units per kilogram of body weight) and the soleus (1/12, 1 unit per kilogram of body weight). Moreover, 3/12 children also received BoNT-A injections in upper limb muscles (in the pronator teres, thumb adductor and/or thumb opponens, with a total upper limb dosage range of 1–2 units per kilogram of body weight). The BoNT-A dosage was defined by the multidisciplinary clinical CP team based on the clinically observed level of spasticity and the degree of the muscle’s involvement in the pathological gait pattern. As part of the standardized BoNT-A treatment protocol, all children received intensive aftercare, including ankle-foot orthoses and regular physiotherapy, and 11/12 children received serial casting of the lower leg after BoNT-A injection for 7–17 days [33]. Significant increases in body weight (+12%, *p* = 0.002), height (+6%, *p* = 0.012) and fibula length (+4%, *p* = 0.032) were observed at 6 months compared with the baseline data. Population characteristics at the different timepoints are depicted in Table 1, while individual patient characteristics at baseline can be found in Supplementary Table S1.

### 2.2. Qualitative Description of the Muscle Tissue

**Figure 1:**
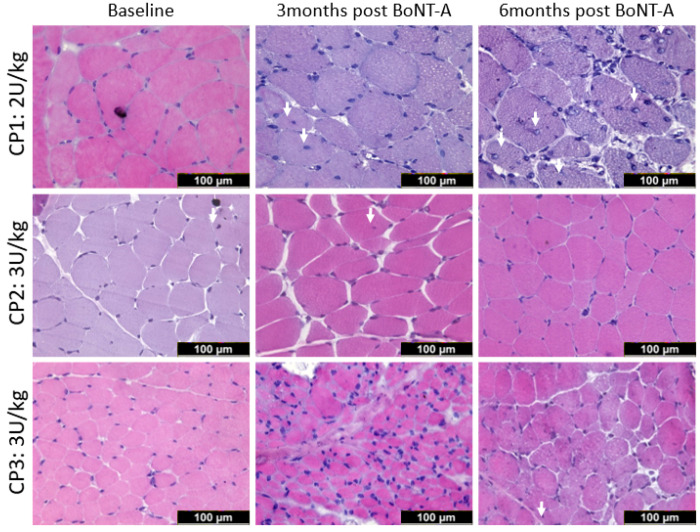
Representative examples of medial gastrocnemius stained with hematoxylin and eosin in three children with CP (CP1, CP2, CP3) at baseline (**left panels**), 3-months post- (**middle panels**) and 6-months (**right panels**) post-BoNT-A injection. The white arrows indicate internal nuclei. See text for details.

Figure 1 shows representative examples (illustrating patterns of response within individuals) of H&E staining of three children with CP, defined at different timepoints. While BoNT-A treatment was associated with muscle structural alterations at 3-months post-injection in comparison with the baseline, the pattern of changes highly differed between children, with CP3 showing severe alterations (Figure 1). These alterations, which were not related to the BoNT-A dose administered, involved an excess of nuclei (CP3) or connective tissue (CP2), the presence of central nuclei (CP1) and variation in fiber size. At 6-months post-BoNT-A, alterations were still observed in CP1 and CP3 but to a lesser extent than after 3 months, while the complete recovery of muscle structure was seen for CP2 (Figure 1).

### 2.3. Fiber Size, Proportion and Coefficient of Variation

**Figure 2:**
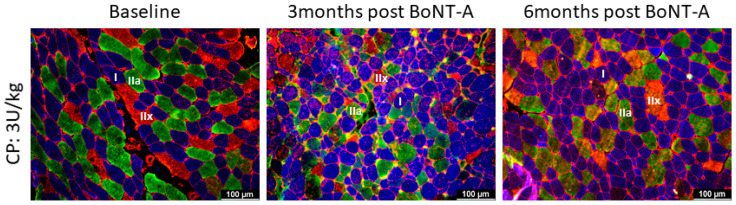
Representative examples of myosin heavy chain staining of the medial gastrocnemius in a child with CP at baseline (**left panel**) and 3 months (**middle panel**) and 6 months (**right panel**) after BoNT-A injection. Type I fibers are stained in blue; type IIa in green; and type IIx and laminin in red. There is great variability in fiber size 3-months post-BoNT-A; such variability is still present at 6 months but to a lesser extent. For quantification of variability, see Figure 4.

**Figure 3:**
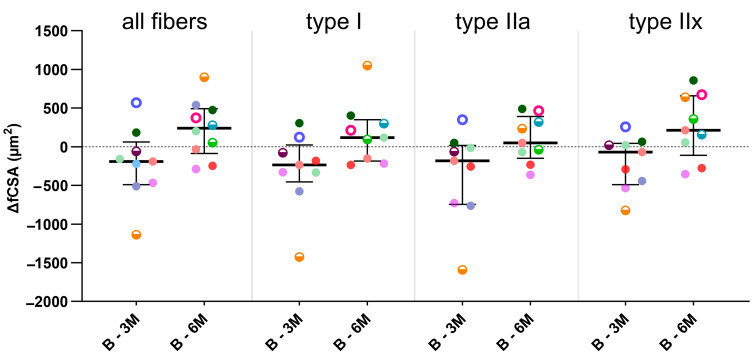
Fiber cross-sectional area differences (ΔfCSAs) between baseline and 3-months post-BoNT-A (B-3M) and between baseline and 6-months post-BoNT-A (B-6M) for all fibers and type I, IIa and IIx fibers. Data are shown as individual values with medians and IQRs. A given symbol and color represent the same child. Open circles represent children who received 1U BoNT-A; half-open circles received 2U BoNT-A; and filled circles received 3U BoNT-A. Statistics-related samples: Wilcoxon signed-rank test.

Typical examples of myosin heavy chain staining of the MG in a child with CP are depicted in Figure 2 at baseline and 3- and 6-months post-BoNT-A treatment. Compared with the baseline, the absolute fiber cross-sectional area (fCSA) of all fiber types decreased by 14% 3 months after BoNT-A injection, mainly because of a reduction in the absolute fCSA of the type IIa fiber (−15%); however, these changes failed to reach statistical significance (Figure 3, Table 2). Six months after BoNT-A injection, there was a non-significant increase in the absolute fCSA of all fiber types (+13%) compared with the baseline. Similar changes were also observed specifically for type IIa (+33%) and type IIx (+61%), but these increases were not statistically significant (Figure 3, Table 2). The normalized fCSA showed similar patterns of changes at each post-BoNT-A timepoint compared with the baseline but without reaching statistical significance (Figure 3, Table 2). The alterations in the fCSA were not related to the dose of BoNT-A.

**Figure 4:**
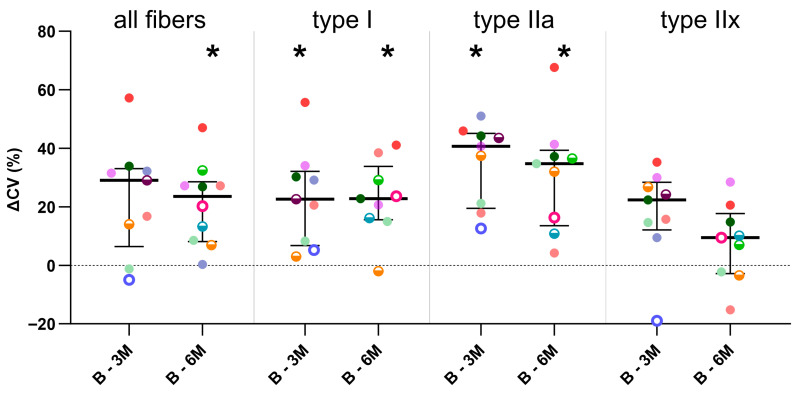
Differences in intra-subject variability in absolute fiber cross-sectional area (coefficient of variation, ΔCV) between baseline and 3-months post-BoNT-A (B-3M) and between baseline and 6-months (B-6M) post-BoNT-A. Data are shown as individual values with medians and IQRs. A given symbol and color represent the same child. Open circles represent children who received 1U BoNT-A; half-open circles received 2U BoNT-A; and filled circles received 3U BoNT-A. Statistics-related samples: Wilcoxon signed-rank test, * *p* < 0.0125 vs. baseline.

Compared with the baseline and independently of the BoNT-A dose administered, the inter-subject variability of the absolute fCSA expressed as CV% significantly increased 3 months after BoNT-A injection for type I (+56%, *p* = 0.032) and type IIa fibers (+137%, *p* = 0.032) and even more so 6 months after BoNT-A (type I: +69%, *p* = 0.04; type IIa: +121%, *p* = 0.032; all fibers: +65%, *p* = 0.020) (Figure 4, Table 2).

A shift toward a slower fiber profile with a relative increase in type I (+6%) and IIa (+24%) proportion and a decrease in type IIx proportion (−45%, *p* = 0.06) was visible 3 months after BoNT-A injection compared with the baseline, but this change did not reach statistical significance. This shift in fiber-type proportion was also clear 6 months after BoNT-A injection, where the type I proportion significantly increased by 17% (*p* = 0.04), while the type IIx proportion decreased by 65% (*p* = 0.032) (Table 2). As a consequence, the relative contribution of the type IIx fibers to the total surface was significantly lower 3 (−44%, *p* = 0.044) and 6 (−64%, *p* = 0.032) months after BoNT-A injection compared with the baseline (Table 2). There were no correlations between the dose of BoNT-A administered and the alterations in fiber proportion.

### 2.4. Capillarization

**Figure 5:**
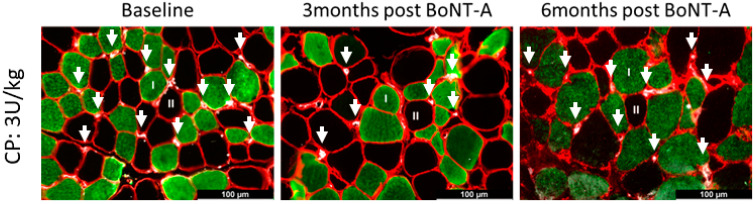
Representative examples of capillary staining using CD31 of the medial gastrocnemius in a child with CP at baseline (**left panel**) and 3 months (**middle panel**) and 6 months (**right panel**) after BoNT-A injection. Type I fibers are stained in green; type II fibers are unstained and appear in black; laminin is stained in red; and white dots represent the capillaries, indicated by an arrow.

Figure 5 shows representative examples of a CD31 staining used to identify capillaries in a child with CP at baseline and 3- and 6-months post-BoNT-A injection. Compared with the baseline, a decrease in the C/F ratio (−26%, *p* = 0.168) and CFD for both type I (−43%, *p* = 0.320) and II (−44%, *p* = 0.0320) fibers was observed at 3-months post-BoNT-A, while the capillary domain (+86%, *p* = 0.576) and heterogeneity of the distribution of the capillaries (+42%, *p* = 0.564) increased, but none of these differences reached statistical significance (Table 2). Six months after BoNT-A injection, these alterations were less pronounced and seemed to normalize toward the baseline values (Table 2). The dose of BoNT-A administered did not impact these data.

### 2.5. Satellite Cell Content

**Figure 6:**
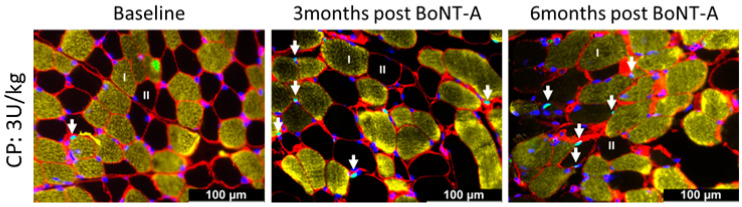
Representative examples of satellite cell staining using PAX7 of the medial gastrocnemius of a child with CP at baseline (**left panel**) and 3 months (**middle panel**) and 6 months (**right panel**) after BoNT-A injection. Type I fibers are stained in yellow, type II fibers are unstained and appear in black, laminin is stained in red, SCs are teal dots (co-localization of PAX7 in green and DAPI in blue) indicated by an arrow.

Figure 6 shows examples of PAX7 staining to identify SCs in a child with CP at baseline and 3- and 6-months post-BoNT-A injection. Compared with the baseline, the number of SCs per 100 fibers seems to increase 3 (+75%, *p* = 0.244) and 6 (+40% *p* = 0.556) months after BoNT-A injection, and this was also the case for the type I fibers (+54%, *p* = 0.168, and +45%, *p* = 0.228, respectively) and type II fibers (+70%, *p* = 0.172, and +50%, *p* = 0.184, respectively) (Table 2). However, none of these differences reached statistical significance. These trends were not related to the dose of BoNT-A administered.

## 3. Discussion

This is the first study examining the time course of BoNT-A’s impact on muscle structure in young, BoNT-A-naïve and ambulant children with CP within a longitudinal perspective. In agreement with the study hypotheses, the results of this pilot study suggest that BoNT-A caused structural alterations in the MG muscle of young, BoNT-A naïve children with CP 3 months after BoNT-A injection in comparison with the baseline condition, but some of these alterations partially normalized, and signs of muscle recovery were observed at 6-months post-BoNT-A independently of the dose administered. These pilot data in particular showed that intra-subject variability in the fCSA was higher 3 months and, even more so, 6 months after BoNT-A injection, especially in the oxidative fibers (types I and IIa). This was accompanied by a non-significant capillary loss at 3 months, which normalized 6 months after BoNT-A injection, while the observed initial shift from a fast to slow muscle profile at 3 months persisted until 6 months after the BoNT-A injection. Evidence of muscle recovery was reflected by the tendency toward increased satellite cell numbers at both timepoints after BoNT-A injection, indicative of satellite cells self-renewing in order to maintain their number after recovery. Importantly, H&E staining revealed that muscle structural alterations in response to a first BoNT-A injection were highly variable between children with CP at both timepoints and were not related to the dose of BoNT-A that was administered. These preliminary data suggest that, 6 months after BoNT-A administration, recovery is still incomplete in the medial gastrocnemius muscles of children with CP.

The structural alterations seen in the MG muscle of the children with CP treated with a single BoNT-A injection in the current study were not as dramatic as the ones reported for animals in whom a loss of contractile material was severe and was replaced by non-contractile fibrotic material. Moreover, these morphological alterations were heterogeneous between the different children with CP, which is in contrast with animal models showing similar muscle alterations in response to BoNT-A. In addition, 6-months post-BoNT-A treatment, recovery was not necessarily present in all of the children with CP in the current study, which differs from data reported for animal models. Recovery status in the present study was not related to the dose of BoNT-A administered to the children with CP. It is, however, important to indicate that all these previously reported severe muscle structural alterations in animal studies were essentially described by the same research group using a healthy female rabbit model with repeated administration of BoNT-A, characterized by a dosage and treatment regimen [11,12,13] that was substantially different from the one used in clinical practice for children with CP [33]. In fact, the structural alterations in the MG muscle observed in the children with CP enrolled in the current study resembled those described in the tibialis anterior muscles of rats after a single injection of BoNT-A [15]. These alterations consisted of decreased fiber size and increased fiber size heterogeneity at 3-months post-BoNT-A, while at 6 months, some central nuclei were present. Similarly, our data are close to the pattern of structural changes in upper limb muscles in children with CP at 4 months to 4 years after BoNT-A compared with BoNT-naïve children with CP, where treated children showed more central nuclei and angular fibers [34]. Likewise, our data overall agree with the mild variation in fiber shape and size and the normal appearance of connective tissue with no evidence of fibrosis or inflammation found in the medial gastrocnemius of BoNT-A-treated children with CP (GMFCS I-III) compared with their vastus lateralis muscles [32]. Previous studies suggest that abnormalities in interstitial connective tissue and/or collagen fibrosis may be correlated with the non-ambulant status of the participants [32,35]. This may explain why connective tissue appears to be normal in the current study involving only ambulant children with CP. Our preliminary data, together with the scarce previous data on muscle structural alterations after BoNT-A administration in children with CP, suggested that muscle morphological alterations were present after BoNT-A in children with CP, but these alterations were less dramatic than the ones reported in animal models. In addition, previous studies have observed signs of regeneration long after BoNT-A administration, indicating a long-lasting process, not yet entirely finalized at 6-months post-BoNT-A treatment. Longer follow-up periods are needed to completely outline this recovery process.

Most animal studies have shown that muscle mass decreased after BoNT-A injection in animal models, irrespective of whether a low or high dose or single or repeated doses were injected [11,12,13,14,15,17], an effect that was still present up to 6-months post-BoNT-A administration with both repeated low and single high doses of BoNT-A [11,15]. Similarly, decreased muscle volume or muscle cross-sectional area was documented with MRI in healthy adults treated with a single dose of BoNT-A up to 12 months after administration [24,25]. This muscle wasting was associated with a reduction in fiber size at 3-months post-high-dose BoNT-A in the rat tibialis anterior [14,15], which persisted for up to 6 months but recovered 12 months after BoNT-A [15]. This was also the case in two healthy humans treated with a single BoNT-A injection in whom the fCSA of the MG was still decreased by 24% 12-months post-BoNT-A compared with saline-treated muscle [24]. By contrast, data for children with CP have been inconsistent, with a decrease in the fCSA being reported in the medial gastrocnemius compared with the vastus lateralis up to 3-years post-BoNT [32], while an increase was found 4 years after BoNT-A in upper arm muscles compared with BoNT-A-naïve children with CP [31]. However, the use of another muscle type for comparison or to combine data from different muscles to assess the effect of BoNT-A could have contributed to these discrepant results. Our preliminary data, collected in the same lower limb muscles within a longitudinal design, indicated a slight but non-significant decrease in the fCSAs of all fibers. This was most obvious for the type IIa MG fibers at 3-months post-BoNT-A injection, followed by a non-significant increase, especially in the type IIx fiber at 6 months. The normalized data on the fCSA, which takes the growth of the child over the 6-month study period into account, showed the same trend. Although the sample size in the current study needs to be enlarged in order to be able to draw a firm conclusion, our data suggested that a transient fiber atrophy developed after the first administration of BoNT-A in the MGs of children with CP, which seems to recover to pre-BoNT-A values by 6-months post-BoNT-A. We believe that the current approach, where the effects of BoNT-A are examined over time in the same child with CP, can provide robust data on the impact of BoNT-A on fiber size and potential recovery when the sample size for this pilot study is enlarged and adequate enough to achieve sufficient power.

As previously reported for the tibialis anterior muscles of rats after a single injection of high-dose BoNT-A, fiber size heterogeneity is present after BoNT-A injection [15]. In fact, increased variability in fiber size has already been shown in the limb muscles of children with CP [36,37,38,39,40], and we recently reported that this was also the case in the MG [41] and ST (unpublished data) muscles of young and ambulant children with CP compared with age-matched typically developing children. We even showed that, in this population, fiber size variability was higher in the BoNT-A-treated children with CP compared with BoNT-A-naïve children with CP [41]. However, these latter studies did not find clear associations between muscle alterations in 2–9-year-old children and clinical symptoms, such as spasticity (classified with the Modified Ashworth scale). Hence, the contributing factors leading to altered microscopic muscle structures need to be further explored. In line with these findings, the preliminary data of the current study showed that fiber size variability, as reflected by CV%, increased 3 months after BoNT-A and persisted at 6 months. This phenomenon was related to higher size variability, mainly in types I and IIa, at both 3- and 6-months post-BoNT-A. These data suggest that the heterogeneity in fiber size, which was already present in the BoNT-A-untreated muscles of children with CP, was further exacerbated after the first BoNT-A treatment, even 6 months after treatment. Importantly, these preliminary data underline that treating children with CP every 3–6 months with BoNT-A might not be recommended, as it may exaggerate fiber size heterogeneity and eventually preclude the proper functioning of the muscle.

Intriguingly, while denervation is expected to induce a fiber shift toward a faster muscle phenotype [42,43], in the current study, a shift toward a slower profile was observed in the MG muscles of the children with CP 6 months after BoNT-A administration. This is unexpected, especially because a loss of type I fibers with type II predominance has been previously observed up to 3-years post-BoNT-A in the MGs of children with CP compared with their vastus lateralis muscles [32]. In this latter study, it was even shown that the number of BoNT-A injections was strongly correlated with the percentage of type II fibers [32]. However, such comparisons with another muscle type should be interpreted with caution. By contrast, no alterations in fiber composition were found in different upper arm muscles of children with CP after 5-months to 4-years post-BoNT-A [34]. Although data regarding fiber type shifts are inconsistent in children with CP, our preliminary data are, however, in agreement with animal studies, which have consistently shown a transition toward a slower muscle phenotype, whatever the dose, as soon as one month after BoNT-A treatment, persisting for up to 18-months post-treatment [14,15,18,44,45]. It has been suggested that chemo denervation might not affect the muscle in the same way as mechanical denervation, and while neural sprouting will occur after BoNT-A treatment, this is not the case after mechanical denervation [18]. It has, therefore, been proposed that the shift toward a slow fiber type after BoNT-A-induced muscle paralysis could be the result of new neurons innervating the muscle, whereas denervation inducing a shift toward a faster fiber type is the result of the reduced frequency and duration of stimulation [18]. In fact, the upregulation of IGF-1 mRNA reported in the quadriceps muscles of rabbits at 6-months post-BoNT-A supports the concept of reinnervation, keeping in mind that IGF-1 is known to play a role in the induction of sprouting and the elongation of regenerating axons [46,47,48]. In addition, the shift toward slower fibers after BoNT-A is also explained by the fact that type I fibers recover better because of early and more extensive sprouting [49]. Reinnervation via neural sprouts post-BoNT-A treatment is, however, a transient phenomenon related to the temporary denervation induced by BoNT-A, such that this reinnervation will only be present until the original neuromuscular junction recovers its initial function. Taken together, our preliminary data support the concept that reinnervation continues 6 months after BoNT-A.

So far, studies examining capillaries in the muscles of children with CP after BoNT-A administration are scarce. One study on upper arm muscles showed an increase in the capillary-to-fiber ratio and in the number of capillaries around the fiber at 5-months to 4-years post-BoNT-A compared with BoNT-A-naïve children with CP [31]. We also recently found a higher capillary-to-fiber ratio in the MGs of children with a BoNT-A treatment history compared with BoNT-A-naïve children with CP [41]. The data of the current pilot study indicate a non-significant reduction in the capillary-to-fiber ratio and capillary fiber density in the MGs of children with CP 3 months after BoNT-A injection followed by a partial recovery at 6 months. Capillary rarefaction after BoNT-A may, therefore, result in impaired muscle fatigue resistance after BoNT-A. This capillary loss tendency at 3 months post-BoNT-A is associated with a non-significant increase in the capillary domain and the heterogeneity of capillary distribution in the MGs of children with CP 3 months after BoNT-A injection, with partial recovery at 6 months. Part of the heterogeneity of capillary spacing is likely related to the increase in fiber size variability seen in the MG post-BoNT-A. Indeed, it has been shown that the heterogeneity of capillary spacing is correlated with variation in fiber size [50,51]. In addition, it is worth mentioning that BoNT-A can induce the release of calcitonin gene-related peptide, a potent vasodilator [52], thereby increasing blood vessel diameter in the muscle during the duration of BoNT-A-induced muscle paralysis [52]. However, it has been shown that blood flow restriction to the muscle limits the degree of atrophy during unloading [53]. This could suggest that the increased blood flow to the muscle after BoNT-A injections is likely to promote muscle atrophy. Our preliminary data suggest that the capillary amount in the MGs of children with CP might transiently be reduced after BoNT-A, but this needs to be confirmed by enlarging the sample size population. However, the potential impact of BoNT-A on blood flow, possibly promoting muscle atrophy, should not be neglected, although it remains to be confirmed.

Despite their relevant role in muscle regeneration, which is expected after BoNT-A chemo denervation, there are currently no data on SC content in the muscles of children with CP after BoNT-A treatment. Our preliminary data show a non-significant increase in the number of SC for both type I and type II fibers in the MG at 3- and 6-months post-BoNT-A in children with CP. This tendency toward increased SC content could suggest muscle recovery. Hence, the SCs seem to react as expected after BoNT-A-injection-induced muscle damage. Additional analyses are, however, needed to confirm the activation of SCs and proper regeneration after BoNT-A in the MG of these children. This is particularly important knowing that our group recently showed that the myogenic capacity of the SC obtained from the MG of young, BoNT-A-naïve children with CP was impaired 6 months after their first BoNT-A injection [54] and persisted for up to one-year post-injection [55], indicating that the stimulus for muscle growth was impaired after BoNT-A. Although our preliminary data suggest that the SC content seems to increase in the MGs of children with CP after BoNT-A, confirmation with a larger sample size is needed to draw firm conclusions. In addition, future research should determine the extent to which the SCs properly function after BoNT-A treatment.

The mechanisms involved in muscle recovery after (repeated) injections with BoNT-A are not well understood. In an experimental nerve injury model, BoNT-A has been shown to accelerate axonal growth and increase the regeneration of myelinated fibers [56] while also releasing neurotrophic factors [57]. Spontaneous recovery from BoNT-A-induced gastrocnemius muscle atrophy in mice is associated with a strong upregulation of the mTOR/S6 kinase signaling pathway with the upregulation of myotrophic factors such as IGF-I and IGF-II [58]. In children with CP, the mechanisms of muscle recovery after BoNT-A administration have not yet been investigated.

Despite many interesting novel findings on the effect of BoNT-A at the microscopic muscular level, showing clear discrepancies with previous findings from animal studies, the data of the current pilot study should be interpreted with caution, as they refer to preliminary data analyses. The sample size needs to be enlarged in order to achieve sufficient power, which would allow us to draw firm conclusions. The recruitment of extra patients at different timepoints is still ongoing. More research is needed on muscle recovery between 6- and 12-months post-BoNT-A injections and on muscle recovery after repeated BoNT-A injection sessions. It should also be noted that BoNT-A may potentially diffuse among neighboring muscles. This could have been the case for one child who received a low BoNT-A dosage in the soleus muscle, which might have partly diffused to the medial gastrocnemius, with a potential impact on the treatment effect. Nevertheless, we believe that our approach, collecting MG biopsies over time from the same child with CP after BoNT-A and the current pilot results, cast more light on the potential impact of BoNT-A on muscle integrity, the time course of these effects and whether partial or complete recovery will occur.

## 4. Conclusions

In conclusion, these preliminary data suggest that BoNT-A induces structural alterations in the MG muscles of children, but these are not as dramatic as the alterations described in animal models. These structural alterations are still present 6 months after BoNT-A injection but with signs of muscle recovery.

## 5. Materials and Methods

### 5.1. Participants and Ethics

Ambulant children with CP, aged 2 to 9 years with gross motor classification system (GMFCS) levels I to III and either unilateral or bilateral involvement, were included in this study. Children were excluded if they were clinically diagnosed with dystonia or ataxia; had received a previous BoNT-A treatment in the MG muscle; underwent orthopedic surgery in the past 2 years; had a history of muscle surgery on the targeted MG; or presented with severe co-morbidities, such as significant cognitive problems. All children with CP received physical therapy and used day and/or night orthoses as part of their standard care. The recruitment of children with CP was conducted through the Cerebral Palsy Reference Center and the Clinical Motion Analysis Laboratory at the University Hospitals of Leuven, Belgium. Prior to participating in this study, written informed consent was obtained from the parents or legal guardians of each participant. The study protocol was approved by the Ethical Committee of the University Hospital of Leuven (S62645).

### 5.2. Botulinum Toxin Administration

BoNT-A, Onabotulinum toxin A (Botox^®^, Allergan, Diegem, Belgium), injections were applied by a pediatric orthopedic surgeon under general (mask) anesthesia, guided by ultrasonography or palpation. A dose of 100 units of BoNT-A was diluted in 5 mL of saline NaCl 0.9%. The BoNT-A dilution was injected at multiple sites under ultrasound control into the gastrocnemius at least two injection sites, but preferably 3 or 4injections sites, with distances of at least 2 cm between injection sites in larger patients; a maximum of 50 units were injected per site. The injections were given in areas with the largest concentration of motor endplates in the muscles selected for treatment, which is three-quarters for the medial head of the gastrocnemius and four-fifths for the lateral head of the gastrocnemius along a reference line from the medial malleolus up to the proximal margin of the medial tibial condyle [8]. A patient-specific dosage for the gastrocnemius muscle was selected based on a clinical examination (assessment of spasticity and range of motion) while the child was awake, based on a 3D gait analysis (where the involvement of medial gastrocnemius spasticity in the gait pattern was assessed), and based on an additional clinical exam that was performed under anesthesia.

BoNT-A is suggested to be more effective when part of an integrated approach, i.e., combined with casting, orthotic wear and physical therapy [59]. Therefore, lower leg casts and removable upper leg casts were applied, as indicated in Supplementary Table S1. The majority of children used day and night orthoses, and all children received intensive physical therapy in the 8–10 weeks following BoNT-A treatment.

### 5.3. Muscle Biopsy Collection

**Figure 7:**
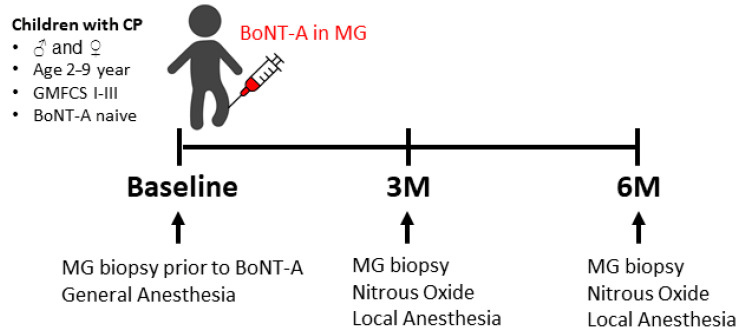
Timeline showing the timepoints at which the biopsies of the medial gastrocnemius (MG) were collected and the type of anesthesia that was used. BoNT-A: botulinum neurotoxin type A; 3M: 3-months post-BoNT-A treatment; 6M: 6-months post-BoNT-A treatment.

Microbiopsies from the muscle belly of the MG were obtained using a disposable core biopsy instrument (Bard^®^ Mission™ Disposable Core Biopsy Instrument 16G, Bard Benelux, Olen, Belgium) under ultrasound guidance, as previously described [60]. A baseline biopsy was collected when the child was under general anesthesia just before BoNT-A (Botox^®^, Allergan) injection (Figure 7). The 3- and 6-months post-BoNT-A injection follow-up biopsies were taken with the child sedated with nitrous oxide (Kalinox^®^) and under local anesthesia: Xylocaine© (lidocaine hydrochloride–anhydrate) 1% (10 mg/mL) with adrenaline (5 µg/mL) (Figure 7). All these procedures were performed at the University Hospital of Leuven. The biopsies were then transferred from the needle, as previously explained [60], to be immediately frozen in liquid-nitrogen-cooled isopentane and stored at −80 °C for further analysis.

### 5.4. Histological Analysis

Sections of 5µm were obtained using a cryostat (−20 °C), transferred onto charged slides (SuperFrost plus, VWR, Leuven, Belgium) and stored at −20 °C for further processing.

#### 5.4.1. Hematoxylin and Eosin Staining

For each muscle sample, a section brought to room temperature was initially rinsed in distilled water and then stained for 1 min using filtered hematoxylin (1.09249, Mayer’s hemalum solution, Sigma-Aldrich, Merck, Hoeilaart, Belgium). Subsequently, the section was briefly rinsed in 0.1% hydrochloric acid for 2 s and then in running tap water for 10 min. Next, the section was stained with eosin (1.09844, eosin Y-solution, Sigma-Aldrich, with 0.2% glacial acetic acid) for 1 min, followed by thorough washing in tap water. Finally, the stained muscle sample was successively rinsed in distilled water; 50% ethanol (EtOH), 70% EtOH, 90% EtOH and 100% EtOH; for 5 min in 1:1 xylene and EtOH; and 5 min in 100% xylene. DPX mounting media (VWR) was applied to cover the muscle section, and a cover slip was placed over it. The sample was dried for 24 h.

#### 5.4.2. Immunofluorescent Staining

Fiber types were identified through myosin heavy chain (MHC) staining; capillaries with CD31; and satellite cells (SC) with PAX7 [41,60]. For capillaries and SC staining, slides were first fixed for 10 min in cold acetone and washed. For all staining, slides were blocked with 10% goat serum blocking buffer in 1xPBS (Thermo Fisher Scientific, Dilbeek, Belgium) and washed for 3 × 5 min in 1% PBS. Slides were then incubated overnight at 4 °C with the corresponding primary antibody cocktail (Table 3). After washing, slides were incubated for 1 h with their corresponding secondary antibody cocktail (Table 3) and washed afterward. Both capillary and SC staining were additionally incubated for 1 min with DAPI (D1306, Thermo Fisher Scientific, Dilbeek, Belgium) and washed. All slides were then mounted using ProLong^®^ Gold antifade reagent (Molecular Probes, Thermo Fisher Scientific, Dilbeek, Belgium) [60].

#### 5.4.3. Image Processing and Quantification

Images of the different channels were taken using a fluorescence microscope (Leica DMi8) at 20× magnification for MHC and 40× magnification for capillaries and SC. The different channels were then merged into one image using the LasX software 3.4.2 (Leica). To determine muscle fCSA and proportion, individual fibers were manually outlined with the ImageJ software. For each fiber type, the fCSA was normalized by dividing the absolute fCSA by the squared fibula length to remove the effect of growth on the absolute fCSA with age. Inter-subject variability in the fCSA was determined by calculating the coefficient of variation (CV = (SD/mean) × 100). The contribution of each fiber type to the total surface area was calculated as the relative contribution by dividing the surface area occupied by each fiber type by the total surface area of all fibers. Capillary analysis was performed with the Btablet and AnaTis applications (www.baloh.nl, NL). The following parameters were determined: (a) capillary-to-fiber ratio (C/F): the number of capillaries surrounding each muscle fiber; (b) capillary fiber density (CFD): the number of capillaries/mm^2^ of muscle fiber; (c) capillary domains: the working areas of capillaries; and (d) the heterogeneity of capillary spacing, calculated as the standard deviation of the logarithm of the domain areas (Log SD). The number of SCs and type I and II fibers were counted on the entire section using the ImageJ software with the ObjectJ plugin. Data are expressed as the number of SCs per 100 fibers.

### 5.5. Statistics

Normality was checked using the Shapiro–Wilk test. Because the majority of the data were not normally distributed, all data are presented as medians (interquartile range (IQR)), except where stated otherwise. Bonferroni correction for multiple comparisons was applied per hypothesis for four primary outcome parameters, fCSA, fiber proportion, C/F and number of SC, by dividing the raw *p*-values by 4, resulting in a critical *p*-value of 0.0125 [61]. The Wilcoxon signed-rank test was conducted to determine differences between baseline and 3-months post-BoNT-A injection and between baseline and 6-months post-BoNT-A injection for all parameters.

## Figures and Tables

**Table 1:** Population characteristics at the three timepoints and changes in body mass, height and fibula length at 3 and 6 months after BoNT-A injection compared with the baseline.

	Baseline	3 Months Post-BoNT-A Treatment	6 Months Post-BoNT-A Treatment
N	12	9	12
GMFCS I/II/III	5/4/3	5/3/1	4/5/3
Boys/Girls	7/5	5/4	7/5
Age (years),	3.4 (2.3)	3.6 (2.2)	3.8 (2.3)
Age range (years)	2.5–7.8	2.7–8.0	3.0–8.3
Body mass (kg)	13.8 (5.0)	14.7 (3.9)	15.6 (4.3)
Δ body mass:		+6%	+12% **
Height (cm)	94.1 (16.0)	97.2 (14.6)	99.0 (18.2)
Δ height:		+3%	+6% *
Fibula length (cm)	19.3 (4.0)	20.0 (4.25)	20.0 (4.0)
Δ Fibula length:		+4%	+4% *
Uni-/Bilateral	4/8	4/5	4/8
Serial casting post-BoNT-A	7–17 days	N/A	N/A

Data are presented as median (interquartile range). GMFCS: gross motor function classification system. Statistics-related samples: Wilcoxon signed-rank test, * *p* < 0.0125, ** *p* < 0.0025. Percentages (%) at 3- and 6-months post-BoNT-A are expressed relative to the baseline values. Notice that one child with CP, classified as GMFC I at baseline, was classified as GMFCS II at 6-months post-BoNT-A treatment.

**Table 2:** Histological measurements of the medial gastrocnemius at baseline and 3- and 6-months post-BoNT-A injection.

			N	Baseline	3-Months Post	ΔMedian (Δ%)	N	Baseline	6-Months Post	ΔMedian (Δ%)
Fiber size and fiber type distribution	Absolute fCSA (µm^2^)	all fibers	9	1027 (528)	879 (476)	−148 (−14%)	10	981 (548)	1113 (771)	+132 (+13%)
		type I	9	1069 (778)	985 (669)	−84 (−8%)	9	999 (526)	1021 (610)	+22 (+2%)
		type IIa	9	825 (680)	698 (448)	−127 (−15%)	9	796 (588)	1062 (845)	+266 (+33%)
		type IIx	9	675 (459)	669 (447)	−6 (−1%)	9	675 (615)	1086 (1112)	+411 (+61%)
	Normalized fCSA (µm^2^/cm)	all fibers	9	49 (31)	42 (19)	−7 (−14%)	10	54 (35)	62 (50)	+8 (+15%)
		type I	9	59 (47)	56 (22)	−3 (−5%)	9	55 (20)	55 (24)	0 (0%)
		type IIa	9	52 (46)	38 (22)	−14 (−27%)	9	52 (44)	59 (48)	+7 (+13%)
		type IIx	9	59 (47)	56 (22)	−3 (−5%)	9	55 (20)	55 (24)	0 (0%)
	CV%	all fibers	9	38 (20)	64 (26)	+26 (+68%)	10	36 (11)	59 (14)	+23 (+65%) *
		type I	9	32 (13)	50 (23)	+18 (+56%) *	9	32 (9)	54 (17)	+22 (+69%) *
		type IIa	9	27 (13)	64 (18)	+37 (+137%) *	9	29 (10)	64 (20)	+35 (+121%) *
		type IIx	9	30 (18)	54 (22)	+24 (+80%)	9	34 (18)	44 (20)	+10 (+29%)
	proportion (%)	type I	9	52 (10)	56 (10)	+3 (+6%)	9	52 (11)	60 (14)	+9 (+17%) *
		type IIa	9	26 (8)	32 (30)	+6 (+24%)	9	24 (7)	31 (15)	+7 (+30%)
		type IIx	9	22 (9)	12 (5)	−10 (−45%)	9	24 (8)	8 (7)	−16 (−65%) *
	relative contribution (%)	type I	9	58 (12)	62 (11)	+4 (+7%)	9	55 (12)	63 (15)	+8 (+14%)
		type IIa	9	26 (10)	29 (11)	+3 (+12%)	9	26 (10)	30 (14)	+4 (+17%)
		type IIx	9	16 (7)	9 (4)	−7 (−44%) *	9	19 (7)	7 (8)	−12 (−64%) *
Capillaries	C/F		5	0.74 (0.28)	0.55 (0.34)	−0.19 (−26%)	7	0.55 (0.54)	0.57 (0.33)	+0.02 (+4%)
	CFD	type I	5	412 (142)	235 (151)	−177 (−43%)	7	370 (155)	363 (246)	−7 (−2%)
		type II	5	453 (172)	254 (134)	−199 (−44%)	7	372 (194)	334 (108)	−38 (−10%)
	domain		5	2347 (843)	4375 (2145)	+2028 (+86%)	7	2559 (951)	3048 (1470)	+489 (+19%)
	heterogeneity index		5	0.18 (0.03)	0.26 (0.08)	+0.08 (+42%)	7	0.18 (0.03)	0.21 (0.07)	+0.03 (+17%)
Satellite cells	SC/100 fibers	all fibers	7	4 (4)	7 (5)	+3 (+75%)	8	5 (3)	7 (3)	+2 (+40%)
		type I	7	5 (3)	8 (4)	+3 (+54%)	8	6 (4)	8 (3)	+3 (+45%)
		type II	7	6 (3)	10 (6)	+4 (+70%)	8	6 (4)	9 (6)	+3 (+50%)

Data are presented as median (interquartile range), differences between 3- or 6-month median and baseline median (Δmedian) and percentage changes relative to baseline (%Δ). fCSA: fiber cross-sectional area, CV: coefficient of variation, C/F: capillary-to-fiber ratio, CFD: capillary fiber density, SC: satellite cells. Statistics-related samples: Wilcoxon signed-rank test. *p*-values are shown as adjusted *p*-values. * *p* < 0.0125.

**Table 3:** Primary and respective secondary antibody cocktails with dilution and brand used for immunofluorescent staining.

	Primary Antibodies			Secondary Antibodies		
	Antibody	Dilution	Brand	Antibody	Dilution	Brand
Myosin heavy chain	laminin	1:250	Ab11575, Abcam, Amsterdam, The Netherlands	Alexa Fluor 680	1:500	#A-21076, Invitrogen, Thermo Fisher Scientific, Dilbeek, Belgium
	mhc-I	1:250	BA-F8, DSHB, Iowa, USA	Alexa Fluor 350	1:500	#A-21140, Invitrogen Thermo Fisher Scientific, Dilbeek, Belgium
	mhc-IIa	1:600	SC-71, DSHB, Iowa, USA	Alexa Fluor 488	1:500	#A-21121, Invitrogen, Thermo Fisher Scientific, Dilbeek, Belgium
	mhc-IIx	1:10	6H1, DSHB, Iowa, USA	Alexa Fluor 555	1:500	#A-21426, Invitrogen Thermo Fisher Scientific, Dilbeek, Belgium
Capillaries	mhc-I	1:250	BA-F8, DSHB, Iowa, USA	Alexa Fluor 488	1:500	#A-21141, Invitrog, Thermo Fisher Scientific, Dilbeek, Belgiumen
	cd-31	1:30	Ab28364, Abcam, Amsterdam, The Netherlands	Alexa Fluor 555	1:500	#A-21428, Invitrogen, Thermo Fisher Scientific, Dilbeek, Belgium
	laminin	1:250	Ab11575, Abcam, Amsterdam, The Netherlands	Alexa Fluor 680	1:500	#A-21076, Invitrogen, Thermo Fisher Scientific, Dilbeek, Belgium
Satellite cells	laminin	1:250	Ab11575, Abcam, Amsterdam, The Netherlands	Alexa Fluor 680	1:500	#A-21076, Invitrogen, Thermo Fisher Scientific, Dilbeek, Belgium
	mhc-I	1:250	BA-F8, DSHB, Iowa, USA	Alexa Fluor 594	1:500	#A-21145, Invitrogen, Thermo Fisher Scientific, Dilbeek, Belgium
	PAX7	1:2	PAX7, DSHB, Iowa, USA	Alexa Fluor 488	1:1000	#A-21121, Invitrogen, Thermo Fisher Scientific, Dilbeek, Belgium

## Data Availability

The raw data supporting the conclusions of this article will be made available by the authors without undue reservation.

## Supplementary Materials

The following supporting information can be downloaded at https://www.mdpi.com/article/10.3390/toxins16020069/s1: Table S1: Individual characteristics of the enrolled children, the BoNT-A dosage (botox^®^) and details on casting immediately following BoNT-A.
