# Supplementary material for: Short-Term Effects of Botulinum Toxin-A Injection on the Medial Gastrocnemius Histological Features in Ambulant Children with Cerebral Palsy: A Longitudinal Pilot Study
Source: Toxins (Basel). 2024 Jan 30;16(2):69. doi: 10.3390/toxins16020069 (PMC10891867; doi:10.3390/toxins16020069)
Supplement: Supplementary file 1 [file toxins-16-00069-s001.zip › toxins-2812386-supplementary.pdf]

Supplementary Materials: Short-Term Effects of Botulinum Toxin-A Injection on the Medial Gastrocnemius Histological Features in Ambulant Children with Cerebral Palsy: A Longitudinal Pilot Study

Table S1. Individual characteristics of the enrolled children, the BoNT-A dosage (botox®) and details on casting immediately following BoNT-A. GMFCS: gross motor function classification system, M: Male, F: Female, BoNT-A: botulinum neurotoxin type-A, 3M biopsy: biopsy 3 months after BoNT-A injection, NA: notavailable.

	Age (years)	sex	GMFCS level	involvement	U BoNT-A (botox® per kg body weight)	casting	time	3M biopsy
**1**	2.50	M	II	unilateral	3	Yes	NA	Yes
**2**	2.80	F	II	bilateral	2	Yes	7 days	No
**3**	2.82	M	I	unilateral	3	Yes	14 days	Yes
**4**	3.21	F	I	bilateral	3	Yes	10 days	Yes
**5**	3.25	F	II	bilateral	3	Yes	14 days	Yes
**6**	3.30	M	III	bilateral	1	Yes	10 days	No
**7**	3.38	M	I	unilateral	3	no	--	Yes
**8**	4.53	M	I	unilateral	1	Yes	14 days	yes
**9**	4.98	F	III	bilateral	2	Yes	14 days	Yes
**10**	5.12	M	III	bilateral	2	Yes	14 days	No
**11**	5.60	F	I	bilateral	3	Yes	17 days	Yes
**12**	7.82	M	II	bilateral	2	Yes	15 days	Yes
